# Diagnosing Severe Falciparum Malaria in Parasitaemic African Children: A Prospective Evaluation of Plasma *Pf*HRP2 Measurement

**DOI:** 10.1371/journal.pmed.1001297

**Published:** 2012-08-21

**Authors:** Ilse C. E. Hendriksen, Juliet Mwanga-Amumpaire, Lorenz von Seidlein, George Mtove, Lisa J. White, Rasaq Olaosebikan, Sue J. Lee, Antoinette K. Tshefu, Charles Woodrow, Ben Amos, Corine Karema, Somporn Saiwaew, Kathryn Maitland, Ermelinda Gomes, Wirichada Pan-Ngum, Samwel Gesase, Kamolrat Silamut, Hugh Reyburn, Sarah Joseph, Kesinee Chotivanich, Caterina I. Fanello, Nicholas P. J. Day, Nicholas J. White, Arjen M. Dondorp

**Affiliations:** 1 Mahidol-Oxford Tropical Medicine Research Unit, Faculty of Tropical Medicine, Mahidol University, Bangkok, Thailand; 2 Centre for Tropical Medicine, Churchill Hospital, University of Oxford, Oxford, United Kingdom; 3 Mbarara University of Science and Technology and Epicentre Research Base, Mbarara, Uganda; 4 Menzies School of Health Research, Casuarina, NT, Australia; 5 National Institute for Medical Research, Amani Centre, Tanga, Tanzania; 6 Medical Research Council Laboratories, Banjul, The Gambia; 7 Kinshasa School of Public Health, Kingasani Research Centre, Kinshasa, Democratic Republic of the Congo; 8 Teule Hospital, Muheza, Tanzania; 9 Malaria Control Program, Ministry of Health, Kigali, Rwanda; 10 Kenya Medical Research Institute (KEMRI)–Wellcome Trust Research Programme, Kilifi, Kenya; 11 Hospital Central da Beira, Beira, Mozambique; 12 National Institute for Medical Research, Tanga Medical Research Centre, Tanga, Tanzania; 13 London School of Tropical Medicine & Hygiene, London, United Kingdom; 14 Medical Research Council, London, United Kingdom; University of Melbourne, Australia

## Abstract

**Background:**

In African children, distinguishing severe falciparum malaria from other severe febrile illnesses with coincidental *Plasmodium falciparum* parasitaemia is a major challenge. *P. falciparum* histidine-rich protein 2 (*Pf*HRP2) is released by mature sequestered parasites and can be used to estimate the total parasite burden. We investigated the prognostic significance of plasma *Pf*HRP2 and used it to estimate the malaria-attributable fraction in African children diagnosed with severe malaria.

**Methods and Findings:**

Admission plasma *Pf*HRP2 was measured prospectively in African children (from Mozambique, The Gambia, Kenya, Tanzania, Uganda, Rwanda, and the Democratic Republic of the Congo) aged 1 month to 15 years with severe febrile illness and a positive *P. falciparum* lactate dehydrogenase (pLDH)-based rapid test in a clinical trial comparing parenteral artesunate versus quinine (the AQUAMAT trial, ISRCTN 50258054). In 3,826 severely ill children, Plasmadium falciparum *Pf*HRP2 was higher in patients with coma (p = 0.0209), acidosis (p<0.0001), and severe anaemia (p<0.0001). Admission geometric mean (95%CI) plasma *Pf*HRP2 was 1,611 (1,350–1,922) ng/mL in fatal cases (n = 381) versus 1,046 (991–1,104) ng/mL in survivors (n = 3,445, p<0.0001), without differences in parasitaemia as assessed by microscopy. There was a U-shaped association between log_10_ plasma *Pf*HRP2 and risk of death. Mortality increased 20% per log_10_ increase in *Pf*HRP2 above 174 ng/mL (adjusted odds ratio [AOR] 1.21, 95%CI 1.05–1.39, p = 0.009). A mechanistic model assuming a *Pf*HRP2-independent risk of death in non-malaria illness closely fitted the observed data and showed malaria-attributable mortality less than 50% with plasma *Pf*HRP2≤174 ng/mL. The odds ratio (OR) for death in artesunate versus quinine-treated patients was 0.61 (95%CI 0.44–0.83, p = 0.0018) in the highest *Pf*HRP2 tertile, whereas there was no difference in the lowest tertile (OR 1.05; 95%CI 0.69–1.61; p = 0.82). A limitation of the study is that some conclusions are drawn from a mechanistic model, which is inherently dependent on certain assumptions. However, a sensitivity analysis of the model indicated that the results were robust to a plausible range of parameter estimates. Further studies are needed to validate our findings.

**Conclusions:**

Plasma *Pf*HRP2 has prognostic significance in African children with severe falciparum malaria and provides a tool to stratify the risk of “true” severe malaria-attributable disease as opposed to other severe illnesses in parasitaemic African children.

*Please see later in the article for the Editors' Summary.*

## Abstract 2

Arjen Dondorp and colleagues investigate whether the plasma level of *Plasmodium falciparum* histidine-rich protein 2 can be used to distinguish between severe malaria and other severe febrile illness in African children with malaria.

## Editors' Summary

**Background:**

Malaria is a life-threatening disease caused by parasites that are transmitted to people through the bites of infected mosquitoes. In 2010, malaria caused an estimated 655,000 deaths worldwide, mostly in Africa, where according to the World Health Organization, one African child dies every minute from the disease. There are four *Plasmodium* parasite species that cause malaria in humans, with one species, *Plasmodium falciparum*, causing the most severe disease. However, diagnosing severe falciparum malaria in children living in endemic areas is problematic, as many semi-immune children may have the malaria parasites in their blood (described as being parasitaemic) but do not have clinical disease. Therefore, a positive malaria blood smear may be coincidental and not be diagnostic of severe malaria, and unfortunately, neither are the clinical symptoms of severe malaria, such as shock, acidosis, or coma, which can also be caused by other childhood infections. For these reasons, the misdiagnosis of falciparum malaria in severely ill children is an important problem in sub-Saharan Africa, and may result in unnecessary child deaths.

**Why Was This Study Done?:**

Previous studies have suggested that a parasite protein—*P. falciparum* histidine-rich protein**-**2 (*Pf*HRP2)—is a measure of the total number of parasites in the patient. Unlike the circulating parasites detected on a blood film, which do not represent the parasites that get stuck in vital organs, *Pf*HRP2 is distributed equally through the total blood plasma volume, and so can be considered a measure of the total parasite burden in the previous 48 hours. So in this study, the researchers assessed the prognostic value of plasma *Pf*HRP2 in African children with severe malaria and whether this protein could distinguish children who really do have severe malaria from those who have severe febrile illness but coincidental parasitaemia, who may have another infection.

**What Did the Researchers Do and Find?:**

The researchers assessed levels of plasma *Pf*HRP2 in 3,826 out of a possible 5,425 African children who participated in a large multinational trial (in Mozambique, The Gambia, Rwanda, Tanzania, Kenya, Uganda, and the Democratic Republic of Congo) that compared the anti-malarial drugs quinine and artesunate for the treatment of severe malaria. All children had a clinical diagnosis of severe malaria confirmed by a rapid diagnostic test, and the researchers used clinical signs to define the severity of malaria. The researchers assessed the relationship between plasma *Pf*HRP2 concentrations and risk of death taking other well established predictors of death, such as coma, convulsions, hypoglycaemia, respiratory distress, and shock, into account.

The researchers found that *Pf*HRP2 was detectable in 3,800/3,826 (99%) children with severe malaria and that the average plasma *Pf*HRP2 levels was significantly higher in the 381 children who died from malaria than in children who survived (1,611 ng/mL versus 1,046 ng/mL). Plasma *Pf*HRP2 was also significantly higher in children with severe malaria signs and symptoms such as coma, acidosis, and severe anaemia. Importantly, the researchers found that high death rates were associated with either very low or very high values of plasma *Pf*HRP2: the odds (chance) of death were 20% higher per unit increase in *Pf*HRP2 above a specific threshold (174 ng/ml), but below this concentration, the risk of death increased with decreasing levels, probably because at lower levels disease was caused by a severe febrile disease other than malaria, like septicemia. Finally, the researchers found that in children within the highest *Pf*HRP2 tertile, the chance of death when treated with the antimalarial drug artesunate versus quinine was 0.61 but that there was no difference in death rates in the lowest tertile, which supports that patients with very low plasma PfHRP2 have a different severe febrile illness than malaria. The researchers use mathematical modeling to provide cut-off values for plasma *Pf*HRP2 denoting the proportion of patients with a diagnosis other than severe malaria.

**What Do These Findings Mean?:**

These findings suggest that in areas of moderate or high malaria transmission where a high proportion of children are parasitaemic, plasma *Pf*HRP2 levels taken on admission to hospital can differentiate children at highest risk of death from severe falciparum malaria from those likely to have alternative causes of severe febrile illness. Therefore, plasma *Pf*HRP2 could be considered a valuable additional diagnostic tool and prognostic indicator in African children with severe falciparum malaria. This finding is important for clinicians treating children with severe febrile illnesses in malaria-endemic countries: while high levels of plasma *Pf*HRP2 is indicative of severe malaria which needs urgent antimalarial treatment, low levels suggest that another severe infective disease should be considered, warranting additional investigations and urgent treatment with antibiotics.

**Additional Information:**

Please access these Web sites via the online version of this summary at http://dx.doi.org/10.1371/journal.pmed.1001297.

- A previous small study in PLOS ONE explores the relationship between plasma *Pf*HRP2 and severe malaria in Tanzanian children
- The WHO website and the website of Malaria No More have comprehensive information about malaria

## Introduction

Severe falciparum malaria in children presents a major diagnostic challenge in malaria-endemic countries where a high proportion of children is parasitaemic at any time. A positive malaria blood smear is therefore not specific for severe malaria, and neither are clinical signs, which are similar to those of other severe childhood infections [1]–[3]. Overdiagnosis of falciparum malaria in severely ill children is an important problem in sub-Saharan Africa [4],[5]. Misdiagnosis is associated with increased mortality [6]. Autopsy studies in children dying with “slide-positive” cerebral malaria show an alternative diagnosis in up to 23% of cases [4]. The central pathological process in severe falciparum malaria is sequestration of trophozoite- and schizont-stage–infected erythrocytes in venules and capillaries, which compromise microcirculatory flow to vital organs [7]. The circulating young ring-form parasites do not sequester and therefore do not reflect accurately the sequestered parasite burden. Thus peripheral parasite counts have weak prognostic significance [8],[9], although this can be improved by assessing the stage of development of these peripheral blood parasites or counting the numbers of malaria pigment–containing neutrophils, which reflects recent schizogony [10],[11].

*Plasmodium falciparum* histidine-rich protein 2 (*Pf*HRP2) is a water-soluble protein found inside the malaria parasite and host erythrocyte, and that circulates free or bound to proteins or antibodies in the plasma compartment [12],[13]. *Pf*HRP2 production peaks during the trophozoite stage, and approximately 90% is released during schizont rupture [14]. Since released *Pf*HRP2 is distributed through the total plasma volume, plasma *Pf*HRP2 can be considered a measure of total parasite burden of the preceding 48-hour asexual parasite life cycle [14],[15]. Studies in Asian adults have shown a strong correlation between plasma *Pf*HRP2, disease severity, and outcome [15],[16].

In the current study we assessed the prognostic significance of plasma *Pf*HRP2 in African children with severe malaria and tested the hypothesis that its assessment could distinguish children with “true” severe malaria, in need of urgent antimalarial treatment, from those with non-malarial severe febrile illness and coincidental peripheral blood parasitaemia, in whom alternative diagnoses and additional treatment need to be considered.

## Methods

The study was part of a large multinational trial comparing quinine and artesunate for the treatment of severe malaria in African children (“AQUAMAT,” ISRCTN 50258054), undertaken between October 2005 and July 2010 [17]. Ethics approval was granted by the Oxford Tropical Research Ethics Committee and the countries' ethics review boards. Full details of this trial have been described elsewhere [17]. In brief, children with signs of severe malaria confirmed by a positive *P. falciparum* lactate dehydrogenase (pLDH)-based rapid diagnostic test were included, provided their parents or caregivers gave full written informed consent. Severity was defined by clinical criteria (see Text S1). Patients were excluded if treated parenterally for >24 hours before admission. Patients were randomised to treatment with either parenteral artesunate or quinine. A venous blood sample was taken for peripheral blood slide, haematocrit (Hct), *Pf*HRP2, biochemistry, and acid-base parameters (EC8+ cartridge for the i-STAT handheld blood analyser). Slide reading was performed by expert microscopists at the Mahidol-Oxford Tropical Medicine Research Unit, and parasites/µl was calculated from thin film (count/1,000 RBC×125.6×Hct) or thick film (count/200 WBC×40) [18],[19].

Plasma *Pf*HRP2 was assessed blinded to patient outcomes from freeze-thawed EDTA plasma samples by a commercial sandwich ELISA kit (Celisa, Cellabs, Sydney, Australia), according to the manufacturer's instructions with minor modifications [15]. Pooled reference plasma from 20 subjects with *P. falciparum* parasitaemia >200,000/µl was calibrated with recombinant *Pf*HRP2 standard (kindly provided by D. Sullivan, John Hopkins School of Public Health, Baltimore, Maryland, US) and used to construct standard curves. Concentrations in duplicate plasma dilutions (1/25 to 1/3,125 in PBS/0.01%Tween) were determined according to the linear segment of the standard curve, with re-assay in cases where duplicates differed by more than 50%. Plasma samples for *Pf*HRP2 were received from nine of the 11 “AQUAMAT” research sites in seven countries (Mozambique, The Gambia, Kenya, Tanzania, Uganda, Rwanda, and the Democratic Republic of the Congo). The study site in Ghana did not collect samples, and the samples from Nigeria defrosted during transportation.

### Individual Patient Estimation of Parasite Burden

Estimation of the total body parasite burden from plasma *Pf*HRP2 has been described in detail in Asian adults with severe malaria and requires incorporation of an elimination half-life estimate [15]. This was assessed separately in African children because clearance might be dependent on immunity (antibodies against *Pf*HRP2), which has a higher level in high transmission settings, and *Pf*HRP2 production is parasite strain dependent [20]. Plasma *Pf*HRP2 half-life was assessed in 30 patients from Tanzania from samples taken on admission and after 3 and 7 days following treatment. Separate ethical approval for this sub-study was obtained from the Ethics Committee of the National Institute for Medical Research, Tanzania. These data were analysed using WinNonlin statistical package (Pharsight, Mountain View, California, US). Individual *Pf*HRP2 concentration-time curves were fitted according to a first-order elimination model. From this, a mean (95%CI) plasma elimination half-life (t_½_) was estimated as 1.10 (0.91 to 1.29) days, or 0.55 erythrocytic cycles. Half-life was not significantly different between treatment arms, and was not correlated with renal function (estimated by blood urea nitrogen [BUN]). A parasite multiplication factor of 3 immediately before peak parasitaemia was assumed, based on in-vitro and Saimiri monkey studies of African parasite strains causing severe malaria [21],[22]. Higher multiplication rates were explored in a sensitivity analysis [23],[24]. The formula for total parasite burden is: P_tot_ = 7.3×PfHRP2×(1−Hct)×body weight [kg] ×10^13^, with *Pf*HRP2 in g/L [15]. The differences in the current formula with the one used earlier in adult Asian patients result from the different estimates for plasma *Pf*HRP2 half-life and parasite multiplication rates. The circulating parasite burden was calculated from the peripheral blood: parasites/µl×10^6^×blood volume ( = 0.08×weight [kg]) [15]. The sequestration index was calculated as total parasite burden/circulating burden [25].

### Statistical Analysis

Data were analysed with STATA, version 10 (Stata Corp., Texas, US). Categorical variables were compared between survivors and fatal cases with Chi-squared or Fisher's exact test. Normally distributed or log_10_-normalized variables were compared using a Student's t-test, the remainder by Wilcoxon rank-sum test. For lowest, middle, and highest tertiles of plasma *Pf*HRP2, comparisons were made between peripheral blood parasitaemia, sequestration index, and treatment effect (mortality) following artesunate versus quinine treatment.

**Figure 1:**
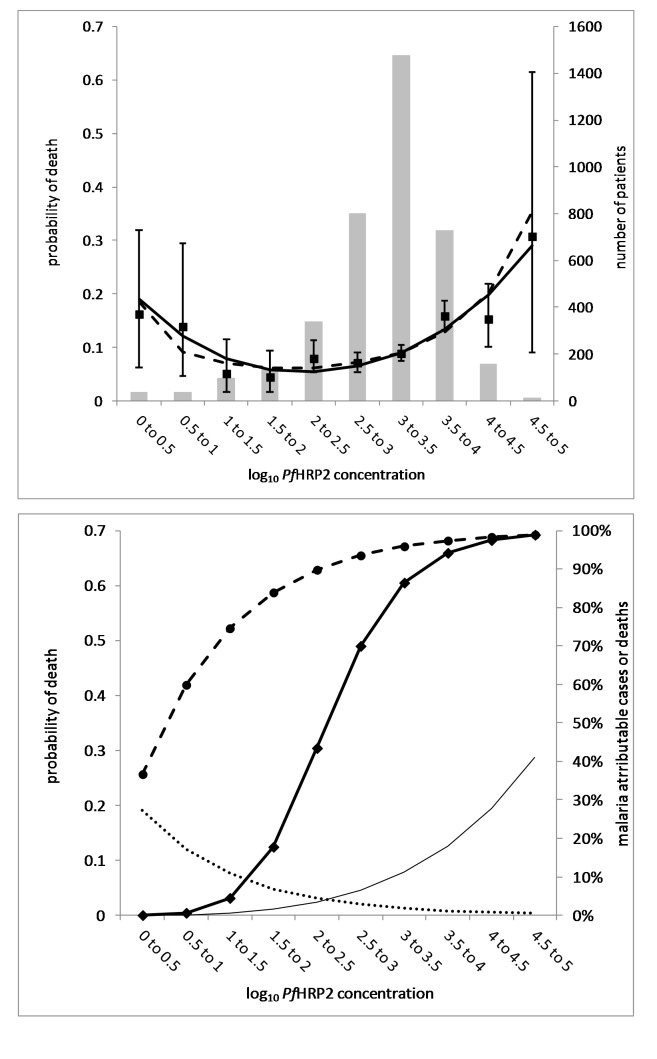
Observed and modelled malaria-attributable mortality and morbidity according to plasma *Pf*HRP2 concentrations. (Top graph) Observed number of patients (grey bars, n = 3,826) and observed probability of death (squares with 95% CI error bars, n = 381) according to *Pf*HRP2 half-log10 strata. The statistical polynomial regression model (dashed line) and the mechanistic model (black line) show the probability of death according to *Pf*HRP2 half-log10 strata. For a detailed description of the mechanistic model see Text S2. (Bottom graph) Malaria-attributable mortality and morbidity according to plasma *Pf*HRP2 concentrations. The curve derived from the mechanistic model (top) describing the relationship between log10 plasma *Pf*HRP2 concentration and probability of death has been deconvoluted in two separate functions: (1) Non[en-dash]malaria-attributable probability of death (dotted line, left axis), which describes the negative exponential probability of dying from non-malaria illness with increasing plasma *Pf*HRP2 concentrations, at a constant *Pf*HRP2 independent case fatality rate of 30%. (2) Malaria-attributable probability of death (thin solid line, left axis), which describes the exponential increase in the probability of death with increasing plasma *Pf*HRP2 concentration, a measure of total parasite burden, in the patient population with “true” severe malaria. From these deconvoluted functions the proportion of the total number of deaths attributable to “true” severe malaria was derived according to *Pf*HRP2 half-log10 strata (diamonds and heavy solid line, malaria-attributable deaths, right axis). Using the “true” severe malaria case fatality rates per *Pf*HRP2 half-log10 strata, the proportion of “true” severe malaria-attributable cases according to *Pf*HRP2 half-log10 strata was derived (circles and dashed line, malaria-attributable cases).

To determine the prognostic significance of plasma *Pf*HRP2, a logistic regression model was constructed with in-hospital death as the dependent variable and *Pf*HRP2 as the independent variable. Since the risk of death showed a non-linear association with log_10_ *Pf*HRP2 (Figure 1, top), both first- and second-degree fractional polynomial functions were explored to find the optimal fit. A quadratic polynomial function provided the best fit using the likelihood ratio test and by comparison of AUCs (areas under the curve). The regression model was stratified for study site and adjusted for treatment and other established predictors of death, including coma, convulsions, prostration, hypoglycaemia, respiratory distress, shock (combined compensated and decompensated), parasitaemia (/µL), haemoglobin (Hb; g/dL), blood urea nitrogen (BUN; mg/dL), and base excess (BE; mmol/L) [8],[9]. Using a stepwise approach, only covariates that were significant at p<0.01 were retained in the final model. Fit of the final logistic regression model was confirmed using the Hosmer-Lemeshow goodness-of-fit test after ordering the data on predicted probabilities and then regrouping the data into 10 nearly equal-sized groups [26].

Any interaction with transmission intensity regarding associations between plasma *Pf*HRP2 and survival was checked and accounted for if significant. Study sites in Mozambique and The Gambia were defined as low transmission; Rwanda, Tanzania, and Kenya as intermediate; and study sites in Uganda and the Democratic Republic of Congo as high transmission.

### Modelling Malaria-Attributable Mortality Based on Plasma *Pf*HRP2

A mechanistic model was constructed to describe the observed U-shaped relationship between *Pf*HRP2 strata and probability of in-hospital death (Figure 1, top), making the following assumptions: (1) an exponential increase of malaria-attributable mortality with plasma *Pf*HRP2, which describes the right side of the curve in Figure 1 (top): Pr_death|malaria_ = −1+exp(k_1_log*Pf*HRP2^k2^); (2) a probability of severe febrile illness due to non-malaria, which decreased exponentially with increasing log*Pf*HRP2: Pr_non-malaria_ = exp(−k_4_log*Pf*HRP2); (3) a risk of death in patients with non-malaria infection equal to 0.3, independent of plasma *Pf*HRP2: Pr_death|non-malaria_ = 0.3 [5],[27]; and (4) that 20% of all deaths were due to non-malaria illness: Death_non-malaria_/Death_total_ = 0.2 [4]. The number of non-malarial deaths according to *Pf*HRP2 stratum is then given by:

Death_non-malaria_ = Pr_death|non-malaria_×Pr_non-malaria_×Cases_total_ and the number of deaths due to malaria by Death_malaria_ = Pr_death|malaria_×(1−Pr_non-malaria_)×Cases_total_. For more details, see Text S2. The effects of assumptions 3 and 4 were explored in a sensitivity analysis.

## Results

### Patient Characteristics

Of the 5,425 children with pLDH-based rapid diagnostic test RDT confirmed falciparum malaria included in the “AQUAMAT” trial, plasma *Pf*HRP2 was measured in 3,826 patients. *Pf*HRP2 could not be measured in 1,600 (29%) patients because the sample was either not collected or not received in optimal condition. Patients without *Pf*HRP2 data did not differ from the remainder regarding malaria slide positivity rate, geometric mean parasitaemia, or case fatality rate. Baseline clinical and laboratory characteristics according to outcome are summarized in Table 1. Although many clinical and laboratory variables associated with severity differed between survivors and fatal cases, admission parasitaemia did not.

**Table 1:** Demographic, clinical, and laboratory characteristics of children diagnosed with severe falciparum malaria according to outcome.

Characteristic	Survivors (n = 3,445)	Fatal Cases (n = 381)	p-Value
Female sex, n (%)	1,692 (49%)	188 (49%)	0.93
Age, y (median, IQR)	2.7 (1.5–4)	2.3 (1.4–4)	0.055
Fever before enrolment, d, median (IQR)	3 (2–4)	3 (2–4)	0.54
Coma before enrolment, h, median (IQR)	4 (2–8)	5 (3–8)	0.020
**Complications on admission**			
Coma (GCS<11 or BCS<3), n (%)	983 (29%)	247 (65%)	<0.0001
Convulsions, n (%)	1,176 (34%)	186 (49%)	<0.0001
Severe acidosis (BE<−8 mmol/L), n (%)	1,132 (41%)	251 (80%)	<0.0001
Severe anaemia (Hb<5 g/dL), n (%)	841 (29%)	117 (34%)	0.030
Hypoglycaemia, n (%)	317 (9%)	136 (36%)	<0.0001
Respiratory distress, n (%)	466 (14%)	103 (27%)	<0.0001
Shock (compensated & decompensated), n (%)	470 (14%)	100 (26%)	<0.0001
Black water fever, n (%)	126 (4%)	18 (5%)	0.30
Jaundice, n (%)	75 (2%)	16 (4%)	0.014
Hyperparasitaemia, n (%)	778 (25%)	101 (30%)	0.046
**Laboratory assessments**			
*P. falciparum* slide positive	99%	98%	0.088
Parasitaemia, geometric mean (range)	45,008 (0–1,858,880)	39,589 (0–1,252,227)	0.33
Blood urea nitrogen, mg/dL, mean (SD)	15 (11)	23 (16)	0.0001
Haemoglobin, g/dL, mean (SD)	6.9 (2.8)	6.5 (2.9)	0.015
pH, mean (SD)	7.38 (0.11)	7.24 (0.19)	<0.0001
HCO_3_, mmol/L, mean (SD)	17.0 (5.4)	11.3 (5.8)	<0.0001
Base excess, mmol/L, mean (SD)	−8 (7)	−16 (8)	0.0001

BCS, Blantyre coma scale; BE, base excess; GCS, Glasgow coma scale, Hb, haemoglobin.

### Plasma *Pf*HRP2 in Relation to Disease Severity

*Pf*HRP2 was detectable in 3,800/3,826 (99%) patients with severe malaria. A detectable plasma *Pf*HRP2 (geometric mean, 95% CI, 450 ng/mL, 209 to 966 ng/mL) with a negative blood slide result (but positive malaria RDT) was found in 36 (0.9%) children. Geometric mean plasma *Pf*HRP2 (95%CI) in survivors was 1,046 ng/mL (991 to 1,104 ng/mL) versus 1,611 ng/mL (1,350 to 1,922 ng/mL) in fatal cases (p<0.0001, Table 2). There was no heterogeneity by stratification for transmission intensity in the difference of plasma *Pf*HRP2 concentrations between survivors and fatal cases (p = 0.1).

**Table 2:** Plasma PfHRP2 according to clinical and laboratory features of severe malaria.

Parameter	Trait	n	Plasma *Pf*HRP2^a^	p-Value
Outcome	Fatal	381	1,611 (1,350–1,922)	<0.0001
	Surviving	3,445	1,046 (991–1,104)	
Coma (GCS≤10 or BCS≤2)	Yes	1,230	1,193 (1,079–1,320)	0.0209
	No	2,596	1,047 (986–1,111)	
Acidosis (BE<−8 mmol/L)^b^	Yes	1,383	1,494 (1,382–1,614)	<0.0001
	No	1,692	969 (896–1,047)	
Severe anaemia (Hb<5 g/dL)^b^	Yes	958	1,585 (1,458–1,722)	<0.0001
	No	2,306	1,044 (975–1,118)	
Shock^c^	Yes	570	1,193 (1,051–1,355)	0.16
	No	3,256	1,075 (1,016–1,138)

BCS, Blantyre coma scale; BE, base excess; GCS, Glasgow coma scale, Hb, haemoglobin.

a Data are geometric mean (95% CI).

b BE available for n = 3,075 and Hb available for n = 3,264 due to missing i-STAT values.

c Compensated and decompensated shock combined.

Plasma *Pf*HRP2 concentrations in relation to established features of severe falciparum malaria are summarized in Table 2. Plasma *Pf*HRP2 was significantly higher in patients with coma, acidosis, and severe anaemia but not in those with shock.

### Estimated Total Body Parasite Burden

**Figure 2:**
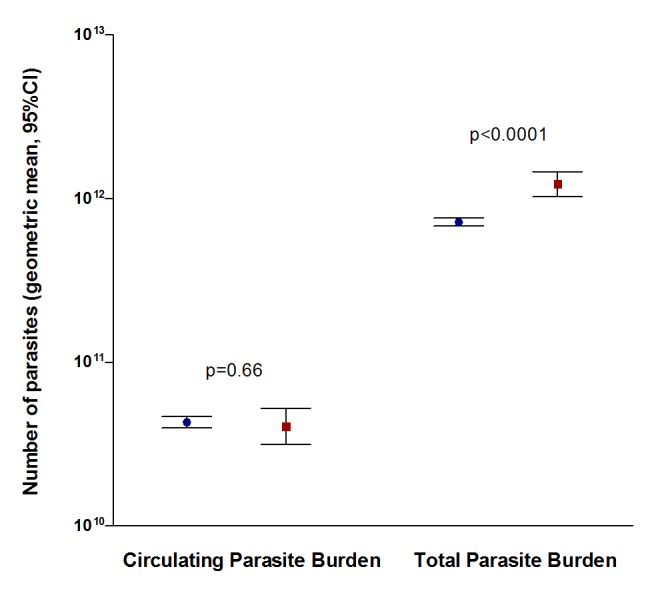
Comparison of circulating parasite burden and total parasite burden between surviving (blue circles, n = 3,070) and fatal (red squares, n = 327) cases. Circulating parasite burden was calculated from the peripheral blood parasitaemia and the total parasite burden was estimated from plasma *Pf*HRP2, including 3,397 patients with both detectable *Pf*HRP2 and malaria parasites on the peripheral blood smear.

Geometric mean (95% CI) *Pf*HRP2-derived total parasite burden was 7.5×10^11^ (7.2×10^11^ to 7.9×10^11^) parasites/body (n = 3,800); this was greater in fatal cases (1.2×10^12^ [1.0×10^12^ to 1.5×10^12^], n = 327) than in survivors (7.2×10^11^ [6.8×10^11^ to 7.6×10^11^], n = 3,070, p<0.0001) (Figure 2). In contrast, the total circulating peripheral blood parasite burden did not differ significantly between survivors and fatal cases (p = 0.66). The geometric mean (95%CI) calculated sequestration index, the ratio of total parasitaemia to circulating parasitaemia was 17 (15 to 18) in survivors, versus 30 (23 to 40) in fatal cases (p = 0.0001). The sequestered parasite burden, calculated by subtracting the circulating parasite burden from the total parasite burden, gave a negative result in 296/3,397 (8.7%) patients. Excluding these patients, the geometric mean (95%CI) total sequestered parasite burden was 7.7×10^11^ parasites/body (7.3×10^11^ to 8.2×10^11^, n = 3,101). A sensitivity analysis varying the multiplication factor and *Pf*HRP2 plasma half-life is shown in Text S3.

### Plasma PfHRP2 and Risk of Death

There was a U-shaped association between plasma *Pf*HRP2 and risk of death with a nadir in case fatality rate at a log*Pf*HRP2 of 2.24 ( = 174 ng/mL; Figure 1, top). In an adjusted logistic regression model, stratified by study site, plasma *Pf*HRP2 was a strong independent predictor of death. Odds for death were 20% higher per unit increase in log*Pf*HRP2 (adjusted odds ratio [AOR] 1.21, 95%CI 1.05 to 1.39, p = 0.009) above a threshold log*Pf*HRP2 value of 2.24 ( = 174 ng/mL). Below this concentration, risk of death increased with decreasing plasma log*Pf*HRP2 (AOR 2.3, 95%CI 1.1 to 5.0; p = 0.03). The final model was adjusted for plasma BE, BUN, coma, convulsions, hypoglycaemia, peripheral blood parasitaemia, and antimalarial treatment (Hosmer-Lemeshow p-value for goodness-of-fit = 0.35).

### Distinguishing Death Attributable to Severe Malaria from Death Attributable to Other Causes

High mortality rates were associated with either very low or very high values of plasma *Pf*HRP2 (Figure 1, top), with the former presumably resulting from a disease other than malaria (including sepsis). The observed case fatalities in the lowest *Pf*HRP2 half log stratum and the higher *Pf*HRP2 strata of ≥3.5–4.0 were both over 15%. A mechanistic model describing the U-shaped correlation between log*Pf*HRP2 stratum and risk of death showed a good fit with the observed data and the statistical model (Figure 1, top). This model was deconvoluted into two separate functions corresponding to non-malaria- and malaria-attributable case fatality rates (Figure 1, bottom). The model showed that below a plasma log*Pf*HRP2 value of 2.24 ( = 174 ng/mL) (derived from the nadir in the polynomial logistic regression model), the probability that death resulted from malaria fell below 50%, corresponding to overall proportions of malaria-attributable severe disease <90% (Figure 1, bottom). In the log*Pf*HRP2 stratum of 3 to 3.5 (1,000 to 3,162 ng/mL) and above, the absolute risk of death due to malaria exceeded 8% with a probability of “true” severe malaria >95% and a probability that a death was caused by severe malaria >85% (Figure 1, bottom). For a sensitivity analysis of the mechanistic model see Text S2.

**Figure 3:**
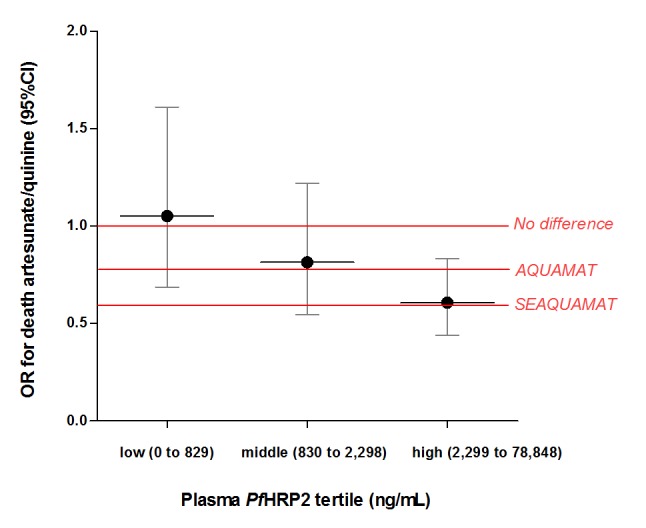
Treatment effect, as odds ratio for death, of artesunate versus quinine. Effect is measured according to plasma *Pf*HRP2 tertiles and compared to the overall treatment effect observed in the AQUAMAT trial [17] in 5,425 African children and in the similar SEAQUAMAT trial [32] in 1,461 (predominantly) adults in Asia.

In patients within the highest *Pf*HRP2 tertile, corresponding to log*Pf*HRP2 ≥3.4 (2,300 ng/mL), the odds ratio (OR) for death in patients treated with artesunate versus quinine was 0.61 (95%CI 0.44 to 0.83, p = 0.0018). In patients in the lowest *Pf*HRP2 tertile, there was no difference in mortalities with an OR for death of 1.05 (95%CI 0.69 to 1.61; p = 0.82, Figure 3). The geometric mean (95%CI) sequestration index, the ratio of total to circulating parasite numbers was 69.8 (60.8 to 80.1) in patients in the highest and 4.6 (4.0 to 5.3) in the lowest *Pf*HRP2 tertile (Table 3).

**Table 3:** Parasite density, Sequestration Index and Treatment effects of artesunate versus quinine according to *Pf*HRP2 tertiles.

*Pf*HRP2 tertiles†	Low (n = 1,115)	Middle (n = 1,154)	High (n = 1,128)
**Plasma ** ***Pf*** **HRP2 (n = 3,397)** ** (range, geometric mean, 95%CI)**	0 to 829218 (201–236)	830 to 2,2981,401 (1,379–1,424)	2,299 to 78,8484,762 (4,598–4,932)
**Parasitaemia (n = 3,397)** ** (geometric mean, 95% CI)**	32,934(28,993 to 37,410)p<0.0001	60,864(53,924 to 68,698)p = 0.041	50,597(44,463 to 57,577)
**Sequestration (n = 3,397)** ** (geometric mean, 95% CI)**	4.6(4.0 to 5.3)p<0.0001	16.9(15.0 to 19.2)p<0.0001	69.8(60.8 to 80.1)
**OR (95% CI) for fatal outcome** ** artesunate versus quinine** ** (n = 3,826)**	1.05(0.69 to 1.61)p = 0.82	0.81(0.54 to 1.22)p = 0.32	0.61(0.44 to 0.83)p = 0.0018

† Tertiles derived from complete *Pf*HRP2 data set (n = 3,826).

## Discussion

This very large prospective study in African children with severe falciparum malaria shows the strong and independent prognostic value of admission plasma *Pf*HRP2 concentration, but not the conventional peripheral blood malaria parasite count. In addition, plasma *Pf*HRP2 was found to be the best immediate measure available to distinguish severe disease caused by malaria from severe febrile illness resulting from another disease with incidental *P. falciparum* parasitaemia. Since *Pf*HRP2 is a measure of total parasite burden, this suggest a critical pathophysiological role played by sequestered parasites in severe falciparum malaria. This is supported by studies which have correlated obstruction of microcirculatory flow in the rectal and retinal circulations to disease severity and outcome, the strong prognostic value of metabolic acidosis in severe malaria, and autopsy studies showing intense sequestration in vital organs [8],[25],[28]–[31].

These results suggest that in areas of moderate or high malaria transmission where a high proportion of children are parasitaemic, admission plasma *Pf*HRP2 can differentiate children at highest risk of death due to severe falciparum malaria from those with likely alternative causes of severe febrile illness. These findings are supported by several observations.

Firstly, plasma *Pf*HRP2 derived total parasite numbers (geometric mean 7.5×10^11^/body) are biologically plausible, and were significantly higher in fatal cases. In contrast, less pathogenic circulating peripheral blood parasite numbers were not correlated with a fatal outcome. The calculated sequestration index was 17 in surviving patients and 30 in non-survivors, which is similar to the median (IQR) sequestration index of 40 (9.9–273.8) calculated directly from post-mortem blood vessel counts in 50 Thai and Vietnamese adults who died from cerebral malaria [25].

Second, the U-shaped curve with a nadir at 174 ng/mL describing the relationship between *Pf*HRP2 and risk of death fits with the assumption that with low *Pf*HRP2, death is caused by non-malarial febrile illnesses (including sepsis) which are independent of the low parasite burden, whereas in patients with plasma *Pf*HRP2 above this nadir the probability of death increases with *Pf*HRP2, representing “true” severe malaria with increasing sequestered parasite burdens. The mechanistic model based on these assumptions had a close fit with the observed data. An alternative explanation could be the presence of highly virulent parasite strains causing severe disease independent of a high total parasite burden. However, this would result in a *Pf*HRP2-independent mortality at the left side of the curve and cannot explain the U shape that was actually observed. Assumptions in constructing the mechanistic model included an alternative cause of death in 20% of patients and a risk of death in non-malaria disease of 30%, based on published autopsy and clinical microbiology data [4],[27]. However, the conclusions were not dependent on these assumptions and were robust to the plausible ranges of values defined for the sensitivity analysis.

Third, the treatment benefit of artesunate over quinine was absent in patients in the lowest *Pf*HRP2 tertile, and strongest in the highest tertile (OR 0.61, 95%CI 0.44 to 0.83, p = 0.0018). Since injectable artesunate can benefit only patients with “true” severe malaria, this provides strong supportive evidence that patients with high *Pf*HRP2 do represent this group, and patients with low *Pf*HRP2 do not. The OR of 0.61 in the highest *Pf*HRP2 tertile is remarkably close to the OR of 0.60 (95%CI 0.45 to 0.79) reported in the large SEAQUAMAT trial comparing artesunate with quinine in the treatment of severe falciparum malaria in 1,461 patients in low-transmission settings in Asia [32]. In these epidemiological settings incidental peripheral blood malaria parasitaemia is rare. The diagnosis of severe malaria based on a peripheral blood slide is therefore highly specific, and so the treatment effect of artesunate over quinine is undiluted by non-malarial disease.

Identification of children with slide-positive severe febrile illness but who do not have severe malaria is important for patient management, since overdiagnosis of severe malaria is associated with increased mortality [6]. A low plasma *Pf*HRP2 should prompt investigation of alternative diagnoses including septicaemia, early administration of parenteral broad spectrum antibiotics (if not already routine), and intensive monitoring. Often antibiotics are given only after a disappointing clinical response to antimalarials, which may be too late. High plasma *Pf*HRP2 concentrations should not discourage antibiotic treatment combined with antimalarial treatment, because of the high proportion of concomitant invasive bacterial disease [2]. Patients with high plasma *Pf*HRP2, which indicates “true” severe malaria with a poor prognosis, should be monitored closely, preferentially in a high-dependency or intensive care unit. As a tool in the design of clinical trials, plasma *Pf*HRP2 is substantially better than peripheral blood parasitaemia in assessing the malaria-attributable fractions and defining the group of patients with “true” severe malaria and a high risk of death (Figure S3 in Text S4 and [33]) An alternative tool is the presence of malaria retinopathy, which has been shown to be highly specific for cerebral malaria as confirmed by post-mortem autopsy [4], although this tool does require training and skilled ophthalmoscopy [34],[35]. It has been evaluated for cerebral malaria [36],[37], whereas many patients with severe falciparum malaria present with other syndromes [17]. *Pf*HRP2 can be used in both cerebral and severe non-cerebral malaria. A direct comparison between the two methods is currently underway. Development of a semi-quantitative rapid test for the detection of plasma *Pf*HRP2 with carefully chosen thresholds could be a valuable tool in high transmission settings to distinguish “true” severe malaria from severe non-malarial febrile illness. For example, a plasma *Pf*HRP2 concentration of >1,000 ng/mL (62.1% of cases in our cohort) denotes a probability >95% of “true” severe malaria with an overall case fatality rate of 11.6% (95%CI 10.3 to 12.9). Defining populations with “true” severe malaria and high mortality is thus critical information for clinicians as well as researchers. In contrast, a plasma *Pf*HRP2 concentration <100 ng/mL (8.1% of cases in our cohort) denotes a probability >15% that severe non-malarial illness is the cause of illness, warranting additional investigations.

Limitations of this study include the inherent dependency of the models on certain assumptions. Estimating the total parasite burden from *Pf*HRP2 is sensitive to the assumed parasite multiplication factor. In the current study the multiplication rate was assumed to be 3, based on in vitro data comparing multiplication rates and multiplication potency of parasites obtained from African children compared to Asian adults. The multiplication rate of 8 used in the original model in Asian adults was based on non-immune adult patient data from the era of malaria therapy of neurosyphilis, and comparable information is obviously not available for our patient group. Applying this higher multiplication rate in this study results in an implausibly high estimated total parasite burden. In addition to differences in parasite multiplication rates, the calculated total parasite burden is dependent on the assumed half-life of plasma *Pf*HRP2, which can vary between patients, and on the amount of *Pf*HRP2 released per parasite per cycle, which can vary between strains [15],[20]. A sensitivity analysis of these parameters is shown in Figure S2 in Text S3. The half-life of plasma *Pf*HRP2 in the current study was shorter than observed in adult patients in Southeast Asia (mean 1.1 versus 3.7 days) [15]. This is presumably related to the African setting where malaria transmission is high and immunological factors including high *Pf*HRP2 antibody titres could increase plasma clearance of *Pf*HRP2 [38],[39]. Since variations in the model parameter estimates are applied to the entire patient group, the model renders either pathophysiologically implausible upper (more parasites than the number of circulating red cells) or lower limits (fewer total parasites than the calculated circulating parasitaemia). Actual total parasite numbers can thus be slightly different from the model estimates. However, differences in the calculated total parasite burdens between subgroups do not depend on the choice of these variables, since these variables will affect this value by the same factor in all subgroups. A recent study in Papuan children with falciparum malaria did not show a correlation between *Pf*HRP2 and disease severity [40]. However, children (n = 220) in this study diagnosed with severe malaria appeared to be only moderately ill as reflected by the <1% case fatality rate and low plasma *Pf*HRP2 values (median 456 ng/mL), whereas patients in that study considered to have uncomplicated malaria had lower plasma bicarbonate concentrations as a measure of acidosis than those with severe malaria. In the present study, <1% cases had undetectable plasma *Pf*HRP2 concentrations, despite presence of *P. falciparum* on the blood slide. This could have been caused by genetic variation in *Pf*HRP2 [41], although this polymorphism is thought not to affect the detection by ELISA [42],[43]. Deletions of the *Pf*HRP2 gene have been reported in field isolates from the Amazon region and in a single report from sub-Saharan Africa [44],[45]. However, the incidence of this genotype is thought to be low in parasites causing severe malaria related to reduced parasite fitness [46],[47]. A study sequencing the *Pf*HRP2 gene in parasites from all patients in the current study who had low plasma *Pf*HRP2 concentrations is underway.

In conclusion, admission plasma *Pf*HRP2 provides a tool in areas of moderate and high malaria transmission to distinguish “true” severe *falciparum* malaria from severe febrile illness with incidental malaria parasitaemia. Plasma *Pf*HRP2 concentrations are a valuable prognosticator in African children with severe falciparum malaria.

## Supporting Information

Text S1 Description of enrolment criteria for severe falciparum malaria.
(DOC)

Text S2 The mechanistic model and sensitivity analysis (including Figure S1).
(DOC)

Text S3 Sensitivity analysis of the estimated total parasite burden as a function of parasite multiplication factor and *Pf*HRP2 half-life (including Figure S2).
(DOC)

Text S4 Plasma *Pf*HRP2 and parasitaemia according to outcome (including Figure S3).
(DOC)

## Abbreviations

AOR: adjusted odds ratio
Hct: haematocrit
OR: odds ratio
OR: odds ratio
*Pf*HRP2: *Plasmodium falciparum* histidine-rich protein 2
pLDH: *P. falciparum* lactate dehydrogenase
RDT: rapid diagnostic test
