# Supplementary material for: Diagnosing Severe Falciparum Malaria in Parasitaemic African Children: A Prospective Evaluation of Plasma PfHRP2 Measurement
Source: PLoS Med. 2012 Aug 21;9(8):e1001297. doi: 10.1371/journal.pmed.1001297 (PMC3424256; doi:10.1371/journal.pmed.1001297)
Supplement: Text S1 — Description of enrolment criteria for severe falciparum malaria. (DOC) [file pmed.1001297.s001.doc]

**Text S1 Description of enrollment criteria for severe falciparum malaria**

Patients needed to fulfill at least one severity criteria of malaria:

Coma Blantyre Coma Score ≤ 2 for children less than 2 years of age or

Glasgow Coma Score ≤ 10 for older children.

Prostration Inability to sit unsupported (for children over 6 months of age) or

the inability to drink or breast-feed in younger children.

Convulsions A duration longer than 30 minutes or a frequency of 2 or more in the 24 hours preceding admission.

Compensated shock Peripheral capillary refill time ≥ 3 seconds or the presence of

a temperature gradient with a normal systolic BP (≥70mmHg).

Decompensated shock Systolic blood pressure <70 mmHg.

Severe respiratory distress Nasal alar flaring, costal indrawing/recession or use of accessory muscles, severe tachypnoea.

Severe acidosis Presence of deep breathing.

Hypoglycemia Blood glucose < 3 mmol/L or clinical improvement in the level of consciousness immediately after administration of 10% dextrose.

Anemia Severe pallor combined with respiratory distress.

Black water fever By caretaker history or observation of dark or black urine.

Jaundice Yellow discoloration of the sclera and skin.

Hyperparasitemia Asexual parasitaemia above 10%.
