# Supplementary material for: Diagnosing Severe Falciparum Malaria in Parasitaemic African Children: A Prospective Evaluation of Plasma PfHRP2 Measurement
Source: PLoS Med. 2012 Aug 21;9(8):e1001297. doi: 10.1371/journal.pmed.1001297 (PMC3424256; doi:10.1371/journal.pmed.1001297)

**Text S2 The mechanistic model and sensitivity analysis**

*Description of the mechanistic model*

The mechanistic model of *Pf*HRP2 describing the relationship between plasma *Pf*HRP2 and probability of death caused by ‘true’ severe malaria and probability of ‘true’ severe malaria in patients diagnosed with severe malaria.

The observed data show a U-shaped relationship between the probability of death and plasma *Pf*HRP2 strata (Figure 2A, nadir log_10_ 2.24 (=174 ng/mL). It was assumed that this U-shaped relationship is a composite of two intersecting curves:

1. The right end of the curve at high *Pf*HRP2 concentrations represents cases with ‘true’ severe malaria with an exponential increase of the risk of death with increasing plasma *Pf*HRP2 concentrations. Two functional forms for this curve were evaluated: a simple exponential function (exp(k_1*h)-1) and an exponential of a power of h (exp(k_1*h^k_2)-1). The latter choice provided a better fit with the observed data and was included in the model, given as:

*Pr_death|malaria_*= *-1+exp(k_1_logPfHRP2^k2^)* ***(eq. 1)***, with the total number of ‘true’ malaria cases (d_m_) defined as:

*d_m_*= [ *-1+exp(k_1_logPfHRP2^k2^)*] x S, with S the total number of cases ***(eq. 2).***

2. The left end of the curve represents cases dying from non-malaria illnesses, which is composed of a probability of non-malaria illness decreasing exponentially with increasing plasma *Pf*HRP2 concentration and a fixed probability of death in cases with non-malaria disease. The exponential form was the most simple decay function that provided the closest fit with the observed data, given as:

*Pr_non-malaria_*= *exp(-k_4_logPfHRP2)* ***(eq. 3),*** with a total number of non-malaria cases (d_0_) defined as:

*d_0_* = *exp(-k_4_logPfHRP2)* x S, with S the total number of cases ***(eq. 4),***

The probability to die given that the patient has a non-malaria illness is independent of plasma *Pf*HRP2:

*Pr_death|non-malaria_= k_3_* ***(eq. 5)***

From eq. 3 and 5 it follows that:

*d_o_ = exp(-k_4_logPfHRP2)* x *k_3_* x S, ***(eq. 6)***

This explains why the left side of the curve in Figure 2B declines with increasing *Pf*HRP2, because the proportion of patients with a different disease than severe malaria (who have a *Pf*HRP2 independent risk of death) declines with increasing *Pf*HRP2 levels, so that the risk of death declines too.

Since the total number of deaths D= d_0_ + d_m_ = S x [*-1+exp(k_1_logPfHRP2^k2^)* + *exp(-k_4_logPfHRP2)* x *k_3_* ] , this represents the overall number of deaths and can be used to fit the relationship between *Pf*HRP2 and observed number of deaths. These equations have 4 parameters to estimate (*k1, k2, k3, k4*).

To reduce the number of possible estimates, it was assumed for the model that *k_3_* = 0.3 and that the total number of deaths caused by non-malaria disease is 20% of the total number of deaths in all *Pf*HRP2 strata (eq. 7). These proportions are based on published literature^4, 5, 27^, but were further explored in the sensitivity analysis within a plausible range according to the consensus of the investigators.

*Death_non-malaria_/Death_total_= k_5_ =* 0.2 ***(eq. 7)***

Fitting the parameters *k1-k5* is done by maximizing the log-likelihood (LL) defined as:

With n = the number of *Pf*HRP2 strata and N(x, μ, σ) being the probability density function of the normal distribution, with mean μ and standard deviation σ, evaluated at x.

The fit of the mechanistic model with the statistical model (adjusted logistical regression model, stratified by study site, see methods section) was confirmed by comparing the predicted probability of death from both models. The mean difference in the predicted probabilities (for n=3024, due to missing values from variables in the statistical model) was 0.96% (95% CI 0.49 to 1.43).

In the sensitivity analysis, k3 was fixed to be in the set {0.3, 0.4} and the proportion of all deaths cases that are not malaria, k5, was fixed to be in the set {0.15, 0.2, 0.25}. The six possible combinations were used in the sensitivity analysis and the model was then refit for every pair of k3 and k5.

*The sensitivity analysis*

A sensitivity analysis including the main model assumptions was conducted for the mechanistic model describing the relationship between *Pf*HRP2 stratum and malaria attributable disease and mortality. The non-*Pf*HRP2 dependent risk of death in non-malarial illness was tested for the values of 0.3 and 0.4. In addition, the total proportion of death caused by non-malarial disease was varied from 0.15 to 0.30. Overall the conclusions derived from the model regarding malaria attributable disease and mortality were robust within the chosen range of values.

The variation in the nadir, describing the *Pf*HRP2 value where the risk of death caused by malaria and non-malaria is equal, is within half a log_10_ value of *Pf*HRP2 (Figure S1-A). Taking the most conservative approximation, the risk of death due to malaria falls below 50% with *Pf*HRP2 <100 ng/ml.

In the log *Pf*HRP2 stratum of 3 to 3.5 (1000 to 3162 ng/ml) and above, the probability of ‘true’ severe malaria varied between 93% and 97% and the probability that death was caused by severe malaria varied between 83% and 93% (Figure S1-B).

**Figure S1-A**  **Figure S1-B**


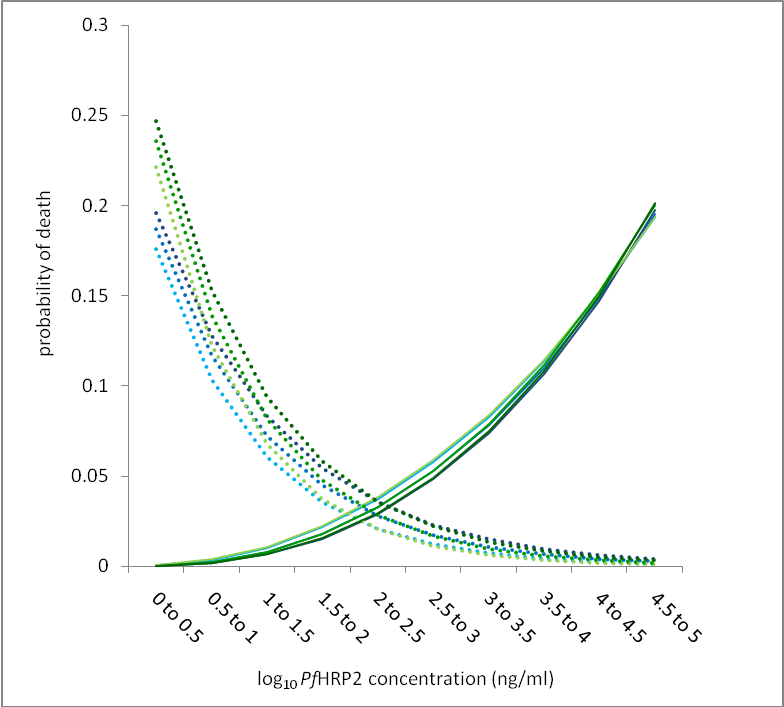

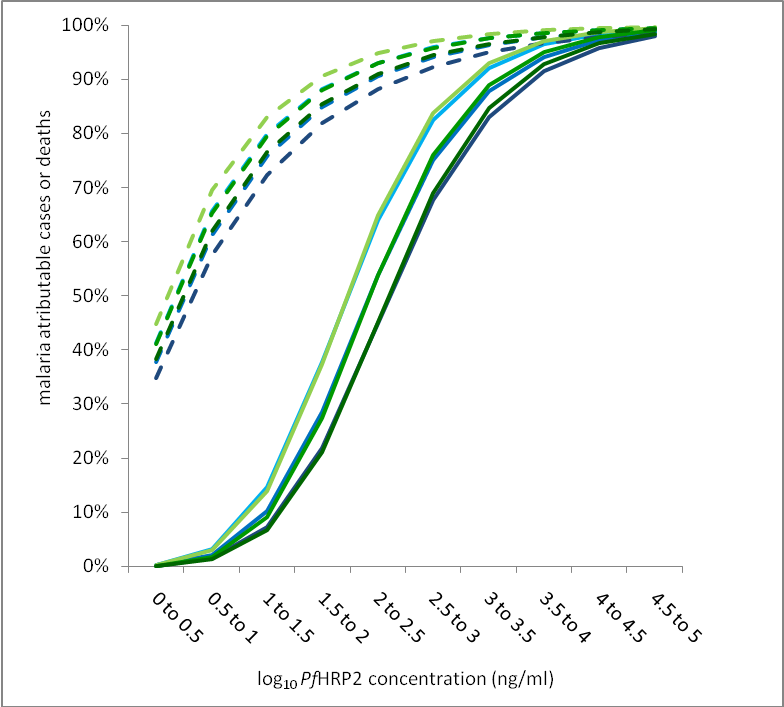


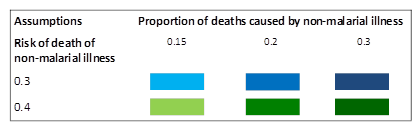

Supplement: Text S2 — The mechanistic model and sensitivity analysis (including Figure S1). (DOC) [file pmed.1001297.s002.doc]
