# Supplementary material for: Diagnosing Severe Falciparum Malaria in Parasitaemic African Children: A Prospective Evaluation of Plasma PfHRP2 Measurement
Source: PLoS Med. 2012 Aug 21;9(8):e1001297. doi: 10.1371/journal.pmed.1001297 (PMC3424256; doi:10.1371/journal.pmed.1001297)
Supplement: Text S3 — Sensitivity analysis of the estimated total parasite burden as a function of parasite multiplication factor and PfHRP2 half-life (including Figure S2). (DOC) [file pmed.1001297.s003.doc]

**Text S3 Sensitivity analysis of the estimated total parasite burden as a function of parasite multiplication factor and *Pf*HRP2 half-life**

The partial rank correlation coefficient between each parameter and the calculated total parasite burden according to the model were 0.57, -0.27 and -0.44 for the parasite multiplication factor, the *Pf*HRP2 half-life and the amount of *Pf*HRP2 secreted per erythrocytic cycle. This indicated that the multiplication factor was the most influential factor affecting the total parasite burden estimate, followed by the amount of *Pf*HRP2 secreted per cycle and the variations in *Pf*HRP2 half-life respectively.

The figures below show the impact of the multiplication factor and the *Pf*HRP2 half-life on the total parasite burden for a patient with a plasma *Pf*HRP2 concentration of 1000 ng/mL, a (population median) haematocrit of 19% and a (population median) bodyweight of 11.2 kg


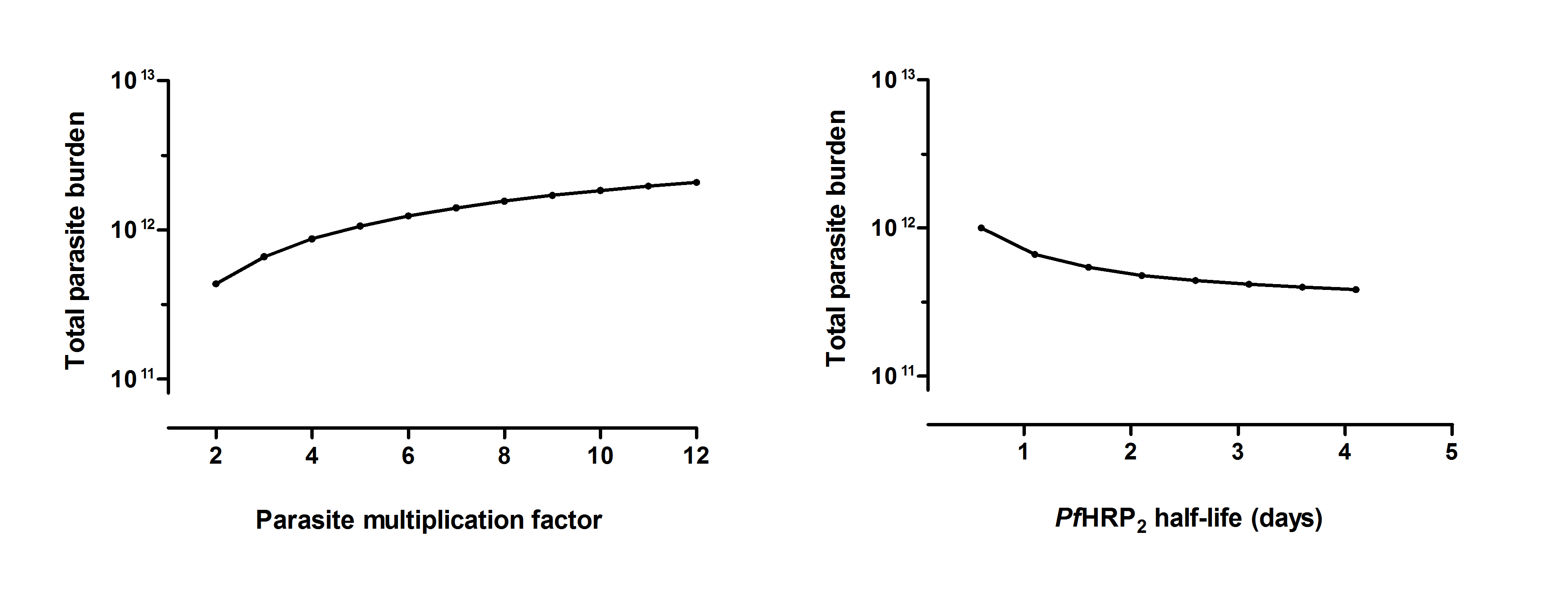


**Figure S2-A and S2-B** Estimated total parasite burden as a function of the parasite multiplication factor or the *Pf*HRP2 half-life using the model as described in the methods. Values chosen for the model parameters were *Pf*HRP2 concentration of 1000 ng/mL, Hct of 19% and bodyweight of 11.2 kg.
