# Supplementary material for: Diagnosing Severe Falciparum Malaria in Parasitaemic African Children: A Prospective Evaluation of Plasma PfHRP2 Measurement
Source: PLoS Med. 2012 Aug 21;9(8):e1001297. doi: 10.1371/journal.pmed.1001297 (PMC3424256; doi:10.1371/journal.pmed.1001297)
Supplement: Text S4 — Plasma PfHRP2 and parasitaemia according to outcome (including Figure S3). (DOC) [file pmed.1001297.s004.doc]

**Text S4 Plasma *Pf*HRP2 and parasitemia by outcome**

**
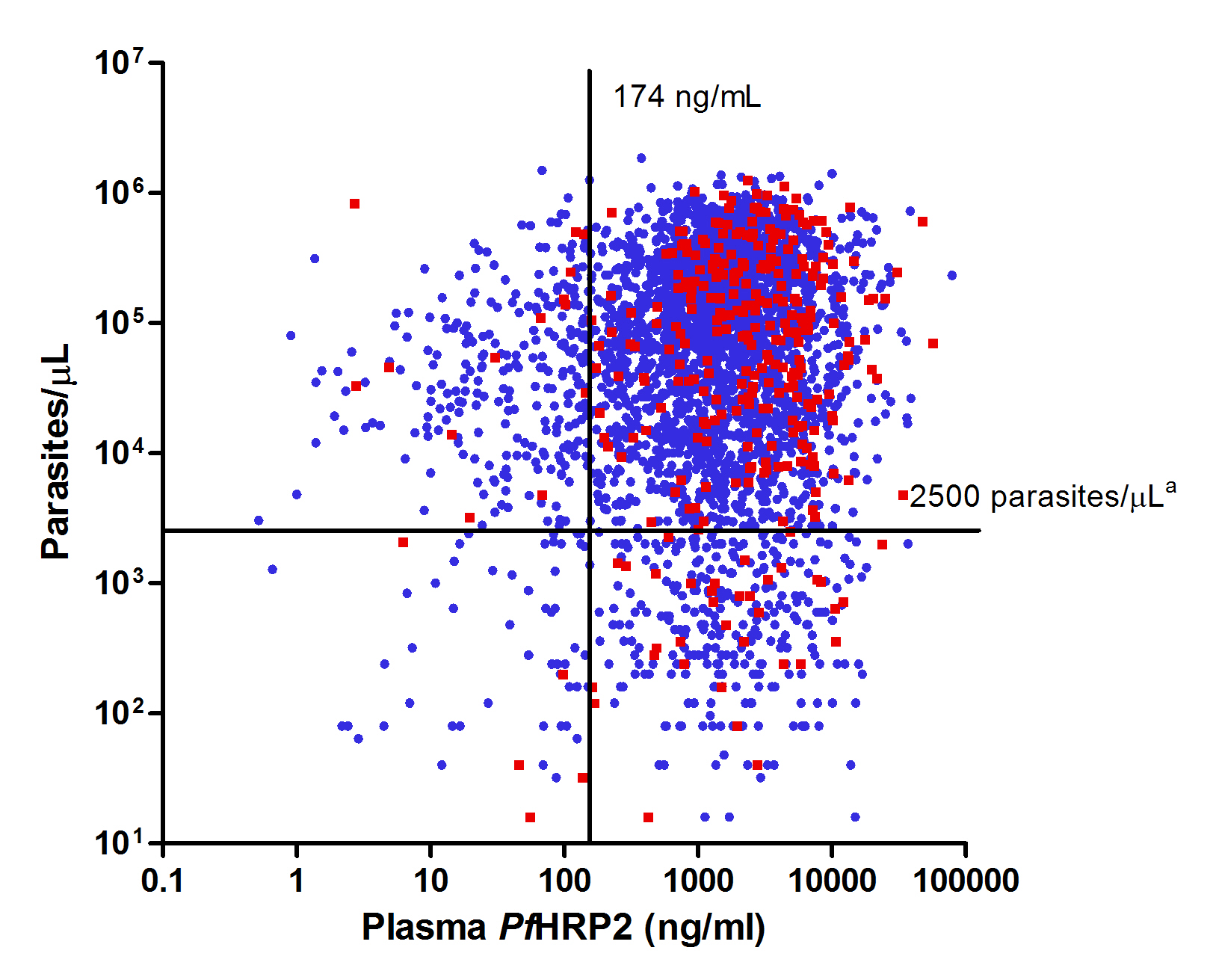
**

**Figure S3**. Scatter plot of parasitemia and plasma *Pf*HRP2 in surviving (●, n=3070) and fatal (■, n=327) cases in patients with both detectable plasma *Pf*HRP2 and malaria parasites seen on the peripheral blood smear.

a Bejon P, Berkley JA, Mwangi T, Ogada E, Mwangi I, et al. (2007) Defining childhood severe falciparum malaria for intervention studies. PLoS Med 4: e251.
